# Anaerobic fungi: effective warriors in lignocellulosic biomass degradation and fermentation

**DOI:** 10.1093/femsec/fiaf108

**Published:** 2025-10-23

**Authors:** Etelka Kovács, Csilla Szűcs, Annabella Juhász-Erdélyi, Zoltán Bagi, Kornél L Kovács

**Affiliations:** Department of Biotechnology and Microbiology, University of Szeged, TTIK, H6726 Szeged, Hungary; HUN-REN Biological Research Center, Szeged, H6726 Szeged, Hungary; Department of Biotechnology and Microbiology, University of Szeged, TTIK, H6726 Szeged, Hungary; HUN-REN Biological Research Center, Szeged, H6726 Szeged, Hungary; Department of Biotechnology and Microbiology, University of Szeged, TTIK, H6726 Szeged, Hungary; Department of Biotechnology and Microbiology, University of Szeged, TTIK, H6726 Szeged, Hungary; HUN-REN Biological Research Center, Szeged, H6726 Szeged, Hungary; Department of Biotechnology and Microbiology, University of Szeged, TTIK, H6726 Szeged, Hungary; HUN-REN Biological Research Center, Szeged, H6726 Szeged, Hungary

**Keywords:** anaerobic digestion, anaerobic fungi, carbohydrate-degrading enzymes, lignocellulose degradation, microbial ecology, rumen microbiology

## Abstract

The significant advancements in understanding the roles of anaerobic fungi (AF) within microbial ecology have opened numerous avenues for biotechnological exploitation, particularly in enhancing the productivity of livestock. The efficient, unique, and complex enzyme systems of AF play a determining role in the metabolic conversion of lignocellulosic plant matter into animal products, such as milk and meat by mammalian herbivores. Mitigation of methane emissions through microbial or dietary strategies in ruminants is a major environmental climate change issue. In turn, controlled management of the interkingdom syntrophic interactions among the eukaryotic AF, prokaryotic bacteria, and archaea can lead to the production of valuable biofuels, (biomethane, biohydrogen, and bioethanol), and organic acids. These products can also serve as building blocks in numerous processes to generate high value chemicals in circular bioeconomy.

## Teaser

Review of the remarkable, unique properties of the unicellular eukaryote anaerobic gut fungi, their role in microbial ecology, potential biotechnological exploitations, and recommendations for further research.

## Introduction

The steadily increasing rate of exploitation of fossil energy carriers and other natural resources accessible on Earth is leading to critical climate changes and resource shortages (Scarlat et al. 2015, Hoogzaad et al. 2020). As a measure to slow down and mitigate the severe consequences, the concept of circular economy (Rogers et al. 2017, Festel 2018, Wiedenhofer et al. 2020) has been developed. The rapid increase of global population and economic development have accelerated the demand for resources worldwide, as demonstrated by escalating geopolitical conflicts. Microorganisms, the most diverse forms of life, may come to our rescue as they can convert materials, which are not digestible by multicellular organisms to components ready to serve multiple levels of the food chain. A deeper understanding of the entire microbiome is necessary, including less abundant anaerobic gut fungi (AGF), members of the phylum Neocallimastigomycota.

Plants and algae capture the solar energy and fix carbon dioxide in the intricate process of photosynthesis. The plant cells convert some of the trapped chemical energy in highly complex lignocellulose molecular constructs and use those as protecting cell wall to sustain their survival (Abramson et al. 2010). This robust and recalcitrant material is composed of three primary polymer families, i.e. cellulose, hemicellulose, and lignin. Cellulose (40%–50% of plant cell walls) is a linear polymer of β(1→4)-linked d-glucose units, whereas hemicellulose (20%–40% of plant cell walls) is a more irregular carbohydrate polymer containing various hexose and pentose derivatives, including glucose, mannose, xylose, and arabinose (Gigli-Bisceglia et al. 2020). Lignin is comprised of cross-linking phenolic precursors (Lebo et al. 2001), which binds primarily to hemicellulose and therefore endows the cell wall with mechanical strength and recalcitrance, which allows the upright position for the plant as a whole (Rao et al. 2023). Plant cell wall decomposition and degradation of the assorted polymeric substances require a large variety of deconstructing enzymes. Microorganisms such as bacteria or fungi are able to produce these enzymes and work synergistically to break down plant cell wall polymers into monomers and oligomers, which can be metabolized by the host animal (Kim et al. 2011).

Herbivores nurture a diverse and abundant microbial ecosystem in their gut that evolved to serve the mutually beneficial coexistence with the host animal. This extended microbial ecology comprises several thousand species of bacteria (Wirth et al. 2012, Henderson et al. 2015, Kakuk et al. 2021), at least 12 genera of ciliate protozoa (Gruninger et al. 2014), 22 genera and over 50 species of AGF (Gruninger et al. 2014, Kakuk et al. 2021, Liang et al. 2023), and ~10 main taxa of methanogenic archaea (Janssen and Kirs 2008, Wirth et al. 2012, Thongbunrod and Chaiprasert 2024), as well as variable collection of mycoplasmas, bacteriophages, archaeophages, and viruses that infect the microorganisms. Drawing a high-resolution picture of the community and especially its functional relationship is challenging, partly because of the redundancy of functions across various taxa (Henderson et al. 2015, Taxis et al. 2015). Research queries and hypotheses also address societal pressures to decrease methane emissions, improve fiber digestibility, feed intake, and feed efficiency, while lowering the risk of milk fat and protein content decline (Firkins and Yu 2015, Aguilar et al. 2018, Hooker et al. 2019, Wiedenhofer et al. 2020). The unique attribute of AGF harboring the herbivores is their extraordinary collection of enzymes for lignocellulose-based biomass decomposition and concomitant hydrogen production. In this condensed review, we focus primarily on the current understanding of AGF, i.e. their taxonomy, lifestyle, position, and role in the microbial ecology and biotechnological potential applications, which convert lignocelluloses into high value products to replace fossil-fuel driven industries (Rubin 2008, Aguilar et al. 2018, Hooker et al. 2019).

## Anaerobic fungi in the microbial ecology of herbivores and elsewhere: who are they?

AGF were first discovered at the beginning of the twentieth century but were not identified as true fungi until the mid-1970s (Orpin 1975, 1977a, Wang et al. 2017, Hanafy et al. 2022). All members of AGF belong to the phylum Neocallimastigomycota, which has only one class (Neocallimastigomycetes) and one order (Neocallimastigales). In the latter, four families (Anaeromycetaceae, Caecomycetaceae, Neocallimastigaceae, and Piromycetaceae) are currently distinguished, based on the classification by Hanafy et al. (2022). Although 22 genera have now been identified, not all of them have found their place in the aforementioned taxa, e.g. *Aklioshbomyces, Khoyollomyces, Astrotestudinimyces*, and *Testudinimyces*. The 22 fungal specified genera are as follows: *Aestipascuomyces, Agriosomyces, Aklioshbomyces, Anaeromyces, Astrotestudinimyces, Buwchfawromyces, Caecomyces, Capellomyces, Cyllamyces, Feramyces, Ghazallomyces, Joblinomyces, Khoyollomyces, Liebetanzomyces, Neocallimastix, Oontomyces, Orpinomyces, Paucimyces, Pecoramyces, Piromyces, Tahromyces*, and *Testudinimyces*. The currently available AGF genome sequences are listed in Table 1. (Youssef et al. 2013, Haitjema et al. 2017, Li et al. 2019, Wilken et al. 2021, Brown et al. 2021, Mondo et al. 2025)

**Table 1.** The sequenced AGF genomes.

Name	Assembly length	Number of genes	Reference
*Anaeromyces robustus* v1.0	71 685 009	12 832	31
*Caecomyces communis* var.churrovis	165 495 782	15 009	30
*Neocallimastix cameronii* var. californiae	193 032 486	20 219	31
*Neocallimastix cameronii* var. constans G3 v1.0	187 118 973	23 663	27
*Neocallimastix cameronii* var. lanati	200 974 851	27 677	29
*Neocallimastix* sp. Gf-Ma3-1 v1.0	209 503 801	28 646	
*Neocallimastix* sp. WI3-B v1.0	206 810 295	28 960	
*Orpinomyces* sp.	100 954 185	18 936	32
*Pecoramyces* sp. F1	106 834 627	17 740	28
*Piromyces finnis* v3.0	56 455 805	10 992	31
*Piromyces* sp. E2 v1.0	71 019 055	14 648	31
*Piromyces* sp. UH3-1 v2.0	84 096 456	16 867	

Mycocosm Portal version:19.390 myco-web-3.jgi.lbl.gov Release Date: 29 August 2025 13:31:46 PST Current Date: 01 September 2025 23:57:25.828 PDT.

Microbiota-related studies in herbivore nutrition focus mainly on bacteria (Wirth et al. 2012, Xu et al. 2021). A very recent breakthrough and large-scale international effort mapped the occurrence and distribution of AGF globally, with geographic, strain and gut-type resolution (Meili et al. 2023, 2024). The wealth of new information about AGF established and confirmed the predominant importance of delicate host–fungi interactions rather than the determining role of feed, location, or lifestyle specificities, i.e. hindgut, pseudoruminant, or ruminant gut anatomy (Solomon et al. 2016, Mura et al. 2019, Hess et al. 2020, Wilken et al. 2020, Jones et al. 2024).

Carbon-rich organic substances are abundant and ubiquitous sources of renewable carbon on Earth. In a broader environmental ecology context AGF participate in recycling industrial, agricultural, and municipal waste into the global carbon and energy cycles (Kim et al. 2011, Wirth et al. 2012, Henderson et al. 2015, Kakuk et al. 2021) in natural ecosystems that may be unrelated to herbivores (Lillington et al. 2020). When finding a suitable anaerobic milieu they usually develop strong symbiotic microbial partnership with other members of the microbial community (Mountfort and Orpin 1994, Edwards et al. 2017, Drake et al. 2021). AGF exist in various forms, assuming sessile and motile planktonic lifestyles (see below). They can adopt various metabolic states under distinct environmental parameters, that is why AGF are difficult to cultivate, particularly in axenic cultures (Mountfort and Orpin 1994). Their incidence and contribution to the microbial activities of the various environmental niches are far from comprehensively explored (Edwards et al. 2017). In addition to mammalian herbivores, they have been detected in natural soils, e.g. peat land (Xue et al. 2021), radioactive landfill site (Lockhart et al. 2006), man-made biogas producing reactors (Kazda et al. 2014), and termite gut (Slaytor et al. 1997, Moreira et al. 2021, Yu et al. 2025). AGF were also found in diverse marine environments such as plankton, estuarine sediments and intertidal sand, in the sea-urchin *Echiocardum cordatum*, and marine iguana *Amblyrhynchus cristatus* (Picard 2017). They seem to be present in anaerobic environments ubiquitously in the biosphere of Earth. Apparently AGF are not plentiful in these environments.

In the rumen, the various taxonomic groups are found in varying abundances depending on diverse environmental ecology factors, i.e. the diet, the feeding regime, physiological condition, and type of the host animal, etc. The current consensus indicates 10^10^–10^11^ bacterial cells ml^−1^, which is predominant in abundance as well as variety. Bacteria comprise about 3000–7000 strains, many of them are still within the “microbial dark matter,” i.e. largely unknown and uncharacterized. The eukaryote AGF and protozoa species are estimated to contribute in the range of 10^3^–10^7^ cells ml^−1^. The rumen Eukaryotes are much larger than their bacteria counterparts so that they can make up an astonishing 50%–55% of the ruminal microbial biomass. Archaea are also part of the unidentified “microbial black matter,” their abundance is in the 10^7^–10^8^ cells ml^−1^ range (Newbold et al. 2015, Beauchemin et al. 2020, Meili et al. 2023, Sanjorjo et al. 2023, Perez et al. 2024).

Unfortunately, the enumeration and evaluation of contribution by traditional cultivation or molecular marker methods is still not well elaborated and validated (Edwards et al. 2017). Methodological inconsistencies, e.g. sampling of the liquid or the solid fractions of rumen content, deviations in DNA and RNA isolation protocols also lead to confusing results. Due to these irregularities, it is difficult to estimate the AGF contribution to overall biological activity relative to the numerically predominating bacterial community members. Kazda et al. (2014) meticulously determined the cell numbers of various taxonomic domains in several biogas plants using advanced microscopic and molecular methods. The rounded numbers indicate 2.03 × 10^8^ eukaryotic fungal cells ml^−1^, 3.5 × 10^8^ methanogenic archaeal cells ml^−1^, and 1.44 × 10^10^ prokaryotic bacterial cells ml^−1^. These findings are in line with the abundances determined in herbivore microbial communities (López-García et al. 2022).

The recent discovery of Neocallimastigomycota molecular markers in fractured igneous deep crust formed in the Miocene age some 20–5 million years ago indicate far reaching evolutionary roles for AGF (Drake et al. 2021). In this epoch of Earth maturity the continental deep subsurface biosphere, including AGF and methanogenic archaea, represented a respectable 2%–19% of Earth’s total biomass (McMahon and Parnell 2014). The early symbiotic relationship between AGF and autotrophic methanogens participated considerably in the formation of methane-rich gases underground, which we exploit as “fossil natural gas” nowadays. The process is also observed in contemporary underground biomethanation sites (Vizzarro et al. 2025, Hu et al. 2025). Taken together, even the available limited and fragmented knowledge suggests that AGF are not some marginal living creatures restricted to the very specific herbivore gut environment. The superficially explored AGF communities are global players, acting commonly in anaerobic environments in symbiotic partnership for millions of years and ready to be employed in diverse biotechnological applications.

## The lifecycle of AGF: how do they live?

The metabolic processes in the rumen are of critical importance in ruminant nourishment and have a major impact on the supply of energy and valuable nutrients to the host (Wirth et al. 2012, Hooker et al. 2019, Xu et al. 2021, Meili et al. 2023, 2024). The predominant foodstuff of herbivores is lignocellulosic biomass. The foraged plant materials are first decomposed by the anaerobic/microaerophilic microbial community of the rumen. During regurgitation, particles travel from the essentially anaerobic rumen into the mouth and are chewed, i.e. mechanically broken down by the ruminant’s teeth in the presence of air. The remaining lignocellulosic material and microorganisms are then sent back to the rumen, where the hydrolysis of the lignocellulosic feed continues under anaerobic conditions. AGF, as well as the other members of the rumen microbiota, must respond to the rapidly changing environmental conditions.

Although Neocallimastigomycota account for about 5%–8% of the herbivorous gut microflora in abundance, they can degrade 20%–50% of the lignocellulosic substrate through their invasive growth and with the help of their amazing collection of unique enzymes (Theodorou et al. 1996, Nicholson et al. 2005, Hartinger and Zebeli 2021).

**Figure 1.**
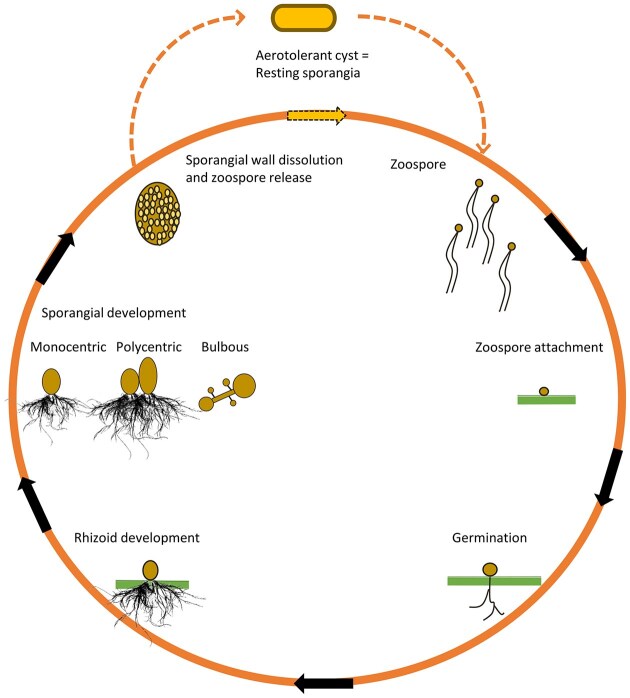
The lifecycle of AGF. For details see the section “The lifecycle of anaerobic gut fungi: how do they live?” The green lines represent the plant cell wall.

The efficacy of lignocellulosic feed digestion by AGF is largely due to their unique lifestyle. The flagellated, free-swimming zoospores find and attach themselves to plant fragments. The process is thought to be governed by chemotactic signals, which are water soluble sugar derivatives from the lignocellulosic substrate (Mountfort and Orpin 1994) (Fig. 1).

The lifecycle (Fig. 1) continues when the encysted zoospores typically germinate on the plant fiber and eventually develop a rhizoidal network into the lignocellulosic tissue. The bulbous forms of AGF do not form long rhizoids, the sporangia launch with very short extensions on the substrate surface. The main zoospore body matures into a thallus and sporangia, which form immobile zoosporangium. The zoospores differentiate and will eventually rupture the mature zoosporangium (Fig. 1) or are released through an apical pore formed at the top of the zoosporangium while the sporangial walls remain intact (e.g. *Pecoramyces ruminantium* strain S4B) (Mountfort and Orpin 1994, Hanafy et al. 2022). The zoospores are equipped with flagella and swim away to colonize new plant material (Bhagat et al. 2023).

In monocentric AGF, the nuclei multiply within the zoosporangium and carry on the genetic information in the mature zoospores. Following the zoospore release, the nucleus depleted vegetative thallus is decomposed and metabolized by the rumen microbiota. In polycentric AGF, the reproductive cycle is less understood and more complex as some nuclei are retained in the rhizoid system. The lifecycle, encompassing motile zoospores, vegetative thallus, and development of new zoospore phases, is short in time. The zoospores have about 10 h to occupy their “new homeland,” degrade the lignocellulosic substrate and metabolize the hydrolysis products. They continue feeding through the rhizoid system in the next 10–20 h while the zoosporangium develops, matures and the next generation of zoospores become ready to be released. Consequently, both zoospore populations and rhizoid thalli should be present continuously in order to grow vigorously, practically indefinitely in a balanced rumen environment (Mountfort and Orpin 1994). The metabolic differences associated with the various life stages were explored in an excellent study recently (Butkovich et al. 2025).

Other segments of herbivore digestive tract, such as the forestomach of kangoroo or the hindgut cecum of horses, offer conditions for AGF that are dissimilar from the rumen. In these challenging environments the AGF abundance is markedly lower than in the rumen. Expressing AGF abundance in thallus forming unit (TFU) per dry matter (DM) g^−1^ measures, the ruminal 1170 × 10^2^ value decreases to 2.18 × 10^2^ in the small intestine but emerges to 288 × 10^2^ in the cecum of young steers (Davies et al. 1993). The pronounced changes indicate an intensive uptake and utilization of microbial biomass in the small intestine and hindgut fermentation and a recovery capability of AGF in the large intestine and feces.

AGF evolved an escape route to survive under unfavorable environmental conditions via forming oxygen resistant and desiccation tolerant, thick walled “resting sporangia,” which have been also mentioned as “survival cysts,” “resistant cysts,” “survival thallus,” or “resistant sporangium” (Davies et al. 1993, Mountfort and Orpin 1994, Hanafy et al. 2022). Little is known about these AGF forms, although they most likely also serve as important dissemination vehicles. Resting sporangia can travel across the deleterious environmental conditions and infiltrate other members of the herbivore herds.

## The metabolism and biochemistry of AGF: how do they function?

### Ruminal fiber degradation by AGF

AGF developed assortments of specific supramolecular tools to deconstruct and metabolize lignocellulosic biomass (Mountfort and Orpin 1994, Solomon et al. 2016, Chen et al. 2024). These will be briefly discussed in this section progressing towards smaller molecular sizes.

#### Rhizoid system

Rhizoids, or structures resembling rhizoids are widespread among the early evolutionary forms of AGF. These structures are similar to the fungal hyphae (Laundon et al. 2020) although the details of rhizoid morphogenesis are less explored than that of hyphae. Carbon resource availability regulates the development of rhizoids and the entire AGF lifecycle. When digestible substrate is detected in the environment, the rhizoids branch densely and the cell growth as well as zoospore formation develops faster. In contrast, under carbon limitation conditions zoospore production is limited, fewer and longer rhizoids develop in search for the lignocellulosic target (Laundon et al. 2020, Bhagat et al. 2023). The fundamental role of AGF rhizoids in the breakdown of lignocellulosic fibers is widely recognized in the rumen by localizing the majority of their impressive array of plant cell wall dismantling enzymes at the rhizoids (Akin and Borneman 1990, Gruninger et al. 2014, Haitjema et al. 2014, Hess et al. 2020, Bhagat et al. 2023). Upon landing on the plant particle surface, the encysted zoospore develops the rhizoids, which delve into the plant tissue (Fig. 1). Recent research demonstrated the efficacy of fungal rhizoids on recalcitrant fibres (Hagen et al. 2021), which may explain the particular significance of AGF when feeding plant fiber forage to ruminants (Himmel et al. 2007). Although both AGF forms attack the ligocellulosic substrate effectively, there is a strategical difference between the filamentous and bulbous forms (Fig. 1). The filamentous forms develop long, branching rhizoid structures to invade the lignocellulosic tissue in depth, while the bulbous forms attach themselves to the surface of the substrate (Hess et al. 2020, Lankiewicz et al. 2022). Accordingly, the filamentous/rhizoid forms, e.g. genera *Neocallimastix* and *Piromyces*, have advantage in decomposing highly lignified tissues (Butkovich et al. 2025). The bulbous AGF, genera *Caecomyces, Cyllamyces*, prefer the physically more fragmented, i.e. already chewed, and thus already surface exposed fodder (Drake et al. 2021).

#### Cellulosomes

**Figure 2.**
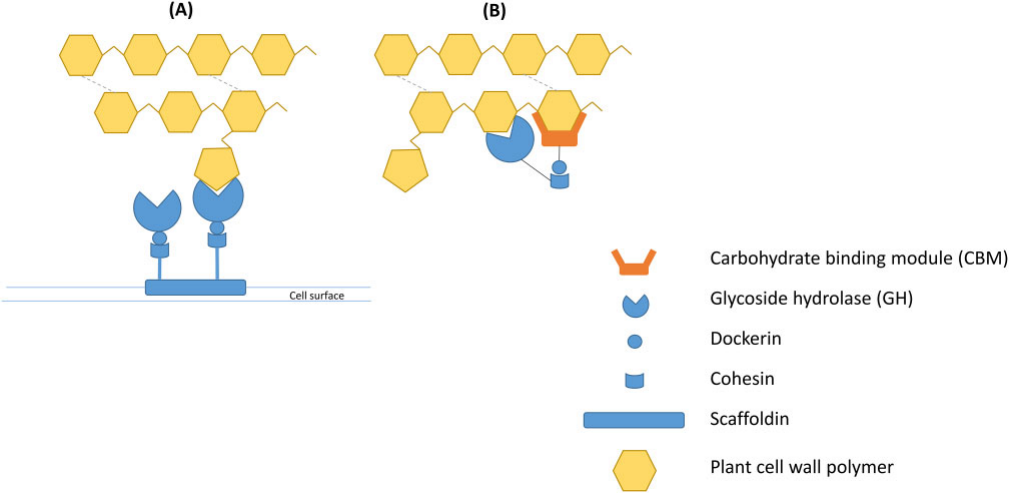
Cartoon models (A) and (B) of polysaccharide decomposition by AGF cellulosome (blue). Note that a large number of various GH enzymes and CBMs interact with the various components of the cellulose and hemicellulose monomers.

The complex structure of lignocellulosic substrates necessitates the development of a sophisticated molecular machinery to degrade, digest, and utilize this recalcitrant material within the relatively short ruminal residence times (Meili et al. 2023) (Fig. 2).

A highly organized multienzyme complex, the cellulosome, is the tool, which accomplishes the effective decomposition (Resch et al. 2013, Haitjema et al. 2014, 2017). The design principles of AGF cellulosomes are strikingly similar to the ones invented by some anaerobic bacteria belonging in the phylum Bacillota (formerly Firmicutes). The main building modules are assembled on the outer surface of the AGF thallus and comprise dockerin, cohesin, catalytic, and scaffoldin protein structures (Gilmore et al. 2015, Ranganathan et al. 2017, Lankiewicz et al. 2022, Brown et al. 2022). Some of the dockerins interact with and secure the entire structure to the AGF external cell surface. A highly organized scaffoldin protein core binds the CBM on the one hand, and serves as a kind of harbor bollard to berth the dockerin–catalytic protein complexes on the other hand (Hsin et al. 2025). Albeit its complexity and several hundreds of kDa size, the cellulosome structure is a flexible, stable and the most efficient molecular structure to handle lignocellulosic biomass. Diverse biotechnological research efforts aim at copying, imitating, and exploiting this remarkable innovation of Nature (Henske et al. 2017). The task is both challenging and rewarding as cellulosomes degrade the lignocellulose substrates up to 50-fold more efficiently than secreted extracellular hydrolases (Hsin et al. 2025).

#### Carbohydrate-active enzymes

The general term, carbohydrate-active enzymes (CAZymes), stands for enzyme families involved in the disintegration of polymeric carbohydrates. The types and structural features of polymeric carbohydrates are varied, hence a massive collection of CAZymes evolved, developed. These are compiled in the incomplete and expanding scientific databases. Up-to-date compilation of the CAZyme classes can be found at the freely available https://www.cazy.org/ page. The publicly available AGF CAZYmes are collected in Table 2 (Drula et al. 2022). Based on the functional differences, five classes of CAZymes are distinguished. Glycoside hydrolases (GH) are enzymes that catalyse the hydrolysis of glycoside bonds. GlycosylTransferases (GT) act in the opposite direction, they catalyse the formation of glycosylic bonds. Polysaccharide lyases (PL) cleave acid-containing polysaccharides with the specific, so called β-elimination mechanism, that is different from the GH and GT mechanism. Carbohydrate esterase (GE) enzymes break the ester bonds present in some polymeric carbohydrates, e.g. in pectin. The enzyme class possessing auxiliary activities comprise redox enzymes that act in conjunction with other CAZymes. In addition to the listed five CAZyme classes, the noncatalytic carbohydrate-binding modules (CBMs) play the important role of anchoring the entire cellulosome assembly to the lignocellulose substrate surface (Bhagat et al. 2023, Hsin et al. 2025, Liggenstoffer et al. 2010) (Fig. 2).

**Table 2.** The list and richness of known CAZYmes in AGF.

Annotation/genomes	*Anaeromyces robustus* v1.0	*Caecomyces communis* var.churrovis	*Neocallimastix cameronii* var. constans G3 v1.0	*Neocallimastix cameronii* var. lanati	*Neocallimastix cameronii* var. californiae	*Orpinomyce*s sp.	*Piromyces* sp. E2 v1.0	*Piromyces finnis* v3.0
CAZy	1609	1984	3362	3452	2480	1778	1894	1385
CBM87	15	24	21	23	22	9	26	19
CBM91	1	1	2	2	2	2	2	1
CBM92						1		
CBM	647	958	1280	1195	844	742	823	490
CE18	13	18	20	21	20	8	21	16
CE	80	83	141	146	137	69	90	65
DOC	444	434	869	991	615	418	302	388
EXPN	14	25	30	32	29	18	18	9
GH5_54						1		
GH13_42	1					1		
GH	261	303	713	752	546	374	472	278
GT	118	126	194	199	177	104	104	100
Myosin_motor	4	4	6	6	6	1	4	4
PL	11	8	86	85	82	30	32	39

Mycocosm Portal version:19.390 myco-web-3.jgi.lbl.gov Release Date:29 August 2025 13:31:46 PST Current Date: 01 September 2025 23:57:25.828 PDT.

These data were obtained after a semimanual curation of protein filtered models sequences by the CAZy team (www.cazy.org) (80).

**Figure 3.**
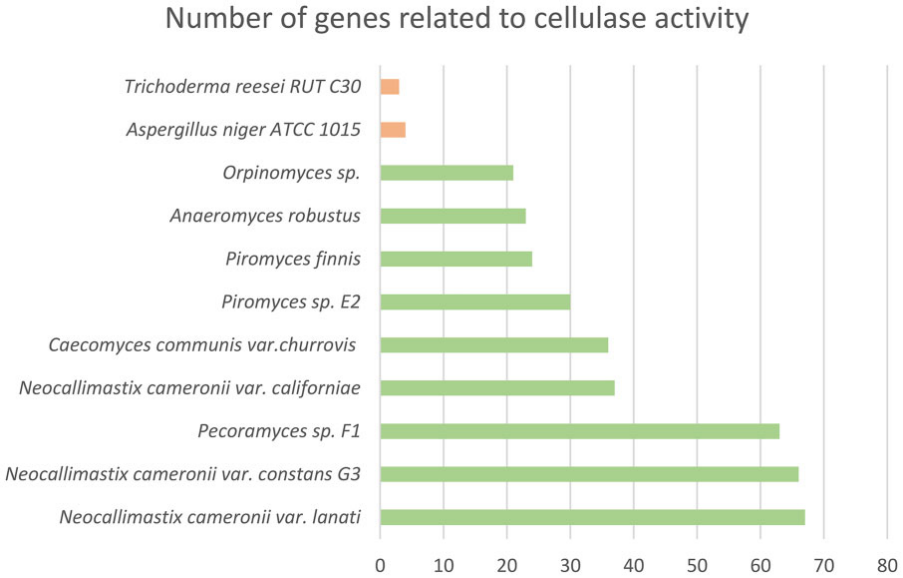
The richness of cellulose decomposing enzymes in filamentous aerobic fungi and anaerobic gut fungi. (39) Mycocosm Portal version:19.390 myco-web-3.jgi.lbl.gov Release Date: 29 August 2025 13:31:46 PST Current Date: 01 September 2025 23:57:25.828 PDT.

Whole-genome sequencing of several AGF species (Kazda et al. 2014, Slaytor et al. 1997, Picard 2017, Perez et al. 2024, Sanjorjo et al. 2023, Beauchemin et al. 2020) revealed that they encode over 16–20 times as many directly cellulose activity related genes as many CAZymes as the filamentous fungi *Trichoderma reesei* or *Aspergillus niger*. Filamentous fungi do not build complete cellulosomes (Fig. 3) (Kovács et al. 2022). This can be correlated with the above mentioned difference in degradation activities between single hydrolases and cellulosome complexes. Nevertheless, filamentous fungi are the most preferred sources of cellulolytic cocktails in bio-based industry (Wilken et al. 2020, Himmel et al. 2007, Lankiewicz et al. 2022) due to their relatively easy enzyme production technology.

#### Lignin degradation by anaerobic fungi

Lignin is a major component of the dry mass of plant cells and the most abundant natural aromatic polymer on Earth (Bomble et al. 2017). With cellulose and hemicellulose fibrils being embedded in a lignin matrix, plant cell walls developed a very robust structural framework (Janusz et al. 2017, Sun et al. 2024). Lignification is obtained by cross-linking reactions of the lignin monomers or by polymer–polymer coupling via radicals produced by oxidases. The result is an array of units linked by carbon–carbon and carbon–oxygen (ether) bonds (Pollegioni et al. 2015). Plants synthesize lignin from *p*-coumaryl, coniferyl, and sinapyl alcohols. The free-radical reaction mechanisms give rise to *p*-hydroxyphenyl (H), guaiacyl (G), and syringyl (S) derivatives that are present in varying ratios in diverse lignins (Lankiewicz et al. 2022, Ralph et al. 2004).

Glucuronoxylan comprises 15%–35% of the secondary cell wall polysaccharides in high lignin biomass, while galactoglucomannan is the main hemicellulose component of secondary cell walls in plants of low lignin content. The latter contains mainly G-lignin, whereas high-lignin plant materials encompass varying ratios of S- and G-lignins (Ek et al. 2009, Suzuki et al. 2012).

Lignin-degrading organisms, such as white rot aerobic fungi, e.g. *Phanerochaete chrysosporium, Trametes versicolor*, and *Pleurotus ostreatus*, thrive in the presence of molecular oxygen (Janusz et al. 2017, Pollegioni et al. 2015, Floudas et al. 2012). The implicated lignin-active enzymes are categorized as laccases, lignin peroxidases, manganese peroxidases, versatile peroxidases, dye-decolorizing peroxidases, other oxidases, and β-etherases (Janusz et al. 2017, Pollegioni et al. 2015). Several aerobic bacteria, belonging in the genera *Pseudomonas, Rhodococcus, Streptomyces, Acinetobacter, Bacillus*, and *Sphingobium*, also produce a subset of these enzymes (Pollegioni et al. 2015, Silva et al. 2021).

The fate of lignin in anaerobic environments remains largely unknown (Meili et al. 2023, Bomble et al. 2017, Young and Frazer 1987) although indirect evidence suggest that certain anaerobic bacteria can break down lignin (Billings et al. 2015, Duan et al. 2016).

In a breakthrough discovery, Lankiewicz et al. (2022) provided straightforward evidence that anaerobic *Neocallimastigomycetes* broke chemical bonds in lignins. 2D-HSQC-NMR data demonstrated the deconstruction of naturally occurring lignin by AGF cultures. The observed anaerobic lignin disruption mechanism by *Neocallimastigomycetes* contrasted with the previously described aerobic processes in both completeness of lignin deconstruction and rate (Lankiewicz et al. 2022). AGF degraded lignin apparently faster than most known bacterial lignin degrading processes but did not reach the rate achieved by white rot fungi (Janusz et al. 2017). Although AGF show ligninolytic potential, their contribution is significant only under anaerobic conditions.

### Bioconversion pathways in AGF

In the previous sections, we summarized how AGF overcome the difficult task of disintegrating the lignocellulosic plant material into smaller molecules effectively (Bauchop and Mountfort 1981, Bhagat et al. 2023, Lima and de Lucas 2022, Saye et al. 2021). Most of the smaller derivatives of polymer breakdown are taken up by the AGF, as well as by anaerobic heterotrophic bacteria present in the microbial ecology landscape, for further utilization via anaerobic fermentation to sustain their life. Eventually, the liberated chemical energy and the fermentation products also drive the metabolic/biosynthetic processes in the animal host for better livestock productivity and health.

The complex substrate/fodder structure requires a multifarious microbial biochemistry to effectively utilize the equally diverse monomeric or oligomeric degradation products. The various microbial functional activities are combined in a fermentation scheme resembling closely the “mixed acids fermentation” biochemistry that is very well known from anaerobic microbiology of Prokaryotes (Bauchop and Mountfort 1981, Panahi et al. 2022). In a simplified version, starting from glucose, the sugar is converted to pyruvate and from there the mixed acids fermentation diverges to yield CO_2_, H_2_, and other small products such as formate, acetate, lactate, succinate, or ethanol. A striking feature among AGF is the highly variable substrate preference and diverse metabolic product spectrum among the members of AGF genera and species (Stabel et al. 2021). The variability may reflect diversity within the AGF taxonomic group and/or the different host–microbiome interaction/molecular communication channels. Extensive future research is needed to explore the fine details of these intimate interactions (Perez et al. 2024, Beauchemin et al. 2020).

### Hydrogenosomes

**Figure 4.**
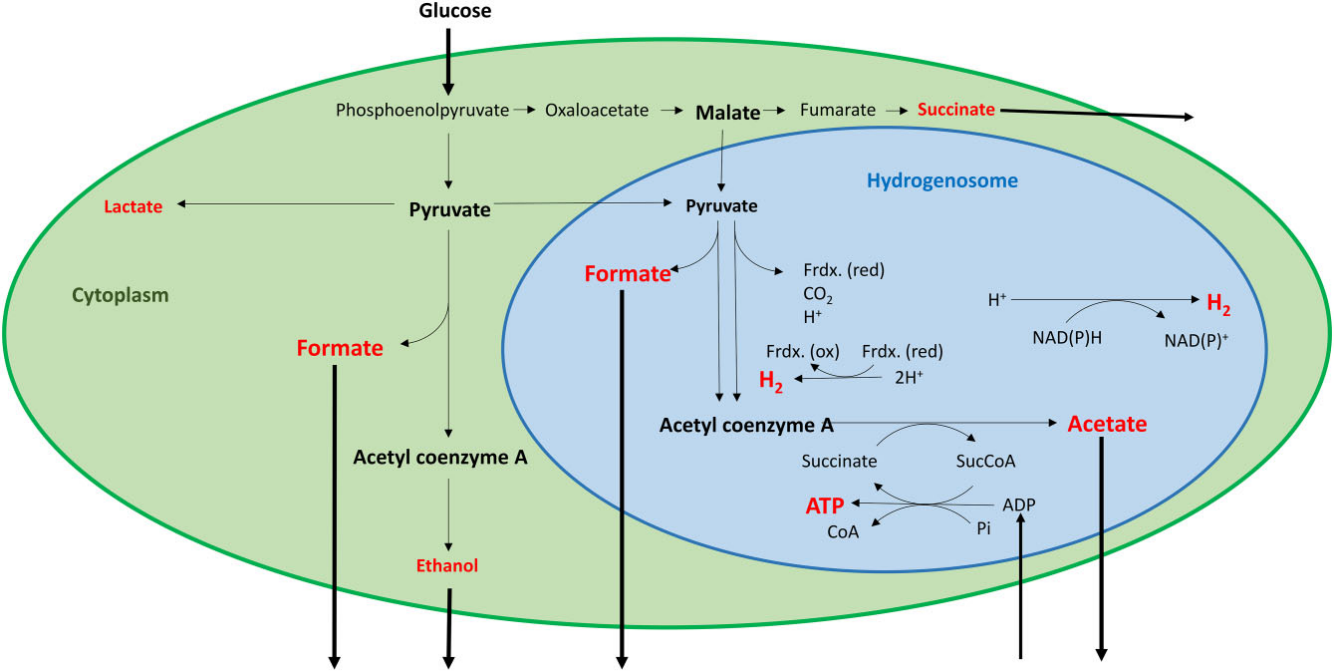
Main sugar fermentation pathways in AGF cytoplasm (green background) and hydrogenosome (blue background). Major fermentation products are highlighted in red.

Hydrogenosomes are membrane-bound organelles in evolutionary distant anaerobic protozoa and fungi. They are unique supramolecular devices evolved primarily to solve the task of chemical energy generation and conservation in the form of ATP (adenosine triphosphate) under anaerobic conditions (Fig. 4).

In the aerobic environment, decomposition of organic molecules takes place in the mitochondria via the well-known tricarboxylic cycle, electron transport, and oxidative phosphorylation using O_2_ as electron acceptor and yielding ATP. Hydrogenosomes have to perform essentially the same task in the absence of O_2_, the widespread terminal electron acceptor (Ma et al. 2022). The enzymology involved is substantially different from the mitochondrial system. Electrons derived from the fermentation of pyruvate are transferred through a chain of redox metalloenzymes including pyruvate ferredoxin oxidoreductase, pyruvate formate lyase, electron carrier ferredoxin, acetate succinyl CoA-transferase enzyme, and various hydrogenases to finally combine with protons and form H_2_ (Stabel et al. 2021, Ma et al. 2022). In bacteria and archaea, an additional hydrogenase system, the so-called confurcating group 3 HydABC complex actively participates in this redox network (Ma et al. 2022). So far, HydABC complex has not been detected in AGF. In order to complete this task successfully, hydrogenosomes are also responsible for antioxidative stress response treatment. The redox metalloenzymes are particularly sensitive to inhibition by reactive oxygen species (ROS). Even very low ROS concentrations may upset the vulnerable cellular redox balance. The reducing capacity of hydrogenosomes contributes as a subcellular equalizer to maintain the redox equilibrium and overall energetic balance (Wilken et al. 2021). The system needs an effective H_2_/reductant safety valve to oxidize the reduced coenzymes, primarily NAD(P)H, and allow the NAD(P)^+^ to return to the anaerobic fermentation cycle. This may involve symbiosis with hydrogenotrophic methanogen Archaea. Cooperation with other H_2_-producing bacteria, e.g. acetogens and/or syntrophic acetate oxidizer bacteria (SAOB), also produce nutrients, which improves amino acid and protein supply for the host animal. However, a sensitive equilibrium should be maintained in the rumen between N-supply and nitrate/nitrite toxicity (Lee and Beauchemin 2014). AGF and their hydrogenosomes may play game changer role in maintaining homeostasis within the AGF cell and in the microbial community. In this context, it is assumed that the sessile, lignocellulose fiber-attached, biofilm forming lifestyle by AGF may assist tolerance to ammonia imbalances (Abera et al. 2025).

It should be mentioned here that additional representatives of microscopic, single celled eukaryotic organisms, Protozoa are also present as important members of the anaerobic environmental ecology landscape of herbivores and other multicellular anaerobic eukaryotes (Martin et al. 1994, Firkins et al. 2020, Bainbridge et al. 2018, Lewis et al. 2018, Newbold et al. 2015). Their distinctive characteristics are the planktonic lifestyle and endosymbiotic engulfment of methanogenic archaea. Their contribution to biomass degradation and utilization is considerable and they frequently harbor unique hydrogenosome organelles, similar to the ones discussed here for AGF. A detailed compendium of the physiology and biochemistry of protozoa is beyond the limitations of this short review and the reader is advised to consult recent outstanding publications (López-Garcia et al. 2022, Priya et al. 2008, Rotterova et al. 2025, Aguilera-Campos et al. 2025, Solomon et al. 2025, Romero et al. 2023, Dey et al. 2004, Davies et al. 1993) for details.

## Biotechnological exploitation potentials of AGF in microbial ecosystems

### Improving ruminal activities

Dey et al. (2004) investigated the effects of *Orpinomyces* sp. culture addition on the daily weight gain, feed intake, growth rate, rumen fermentation, and nutrient digestion in calves of about 10 months of age. A remarkable 15.4% daily weight gain was noted due to more efficient diet utilization. This is astonishing, particularly considering that in the experiment only 160 ml of *Orpinomyces* sp culture, containing 10^6^ CFU ml^−1^ (we presume that the colony forming unit CFU stands for TFU; Davies et al. 1993), were added weekly. The ~100 l volume of the calves’ rumen thus received about 10^10^–10^11^ bacteria/archaea cells ml^−1^ (Perez et al. 2024). The added AGF supplement was diluted roughly 1000-fold, thus contained about 10^3^–10^−4^  *Orpinomyces* sp. CFU/TFU. This AGF population competed in the rumen with an approximately million-fold excess of other microbial cells for the lignocellulosic fodder biomass. The huge prokaryote superiority remains even if the 10-fold size difference and the unknown prokaryote fraction possessing polymer degrading capability are taken into account.

A very similar observation was made when buffalo calves received roughly the same doses of *Orpinomyces* sp. C-14 or *Piromyces* sp. WNG-12 AGF cultures (Tripathi et al. 2007). The fungal inocula were administered every 2 days in this case. Feed digestibility increased by around 10% relative to the control group. In addition, improvements in animal growth rate, body weight gain, and feed efficiency were reported. The digestibility of dry matter, crude protein, neutral detergent fiber, acid detergent fiber, cellulose, and the milk fat were elevated in the AGF-treated animals fed with a wheat straw based diet. AGF apparently boosted ruminants’ production *in vivo* although industrial scale production of live AGF cultures is still an unsolved task for feed additive development.

In an additional *ex vivo* system, the effect of AGF supplementation of a *Piromyces* strain (CN6 CGMCC 14449) to whole crop maize silage anaerobic degradation was investigated (Wang et al. 2019). The *in vitro* tests corroborated the increased lactate, crude protein, and water soluble carbohydrate contents after 30 days.

A geographically distant environment was selected in a detailed study of ruminant AGF (Liang et al. 2023). Grazing yak and cattle herds were examined at high altitude of the Tibet Plateau during a 1-year period. An elevated AGF richness and diversity was found in both the yak and cattle herds in the cold season relative to the warm season. Positive correlation between dry matter, neutral detergent fiber, hemicellulose content, and the AGF abundance was established.

In summary, the limited number of centered scientific investigations call for more and extended studies of the outstandingly effective contribution of AGF in lignocellulose forage utilization. We recommend to use the elaborated classification system developed by Hanafy et al (2022).

AGF and associated anaerobic fermentation cannot fully oxidize to CO_2_ the primary organic carbon source, e.g. lignocellulose. The fermentation products are either energy-rich molecules, e.g. VFAs, or energy-rich electrons, which are stabilized in gaseous H_2_ or as reducing power bound to coenzymes.

As discussed above, the hydrogenosome–cytoplasmic intracellular metabolism equilibrium may direct the energy flow towards either H_2_/formate or biosynthetic pathways. The dynamically changing intracellular ATP status likely determines the ratio between the two pathways in AGF. Understanding the fine details of the mechanism of this energy switch could be a key to managing more efficient ruminal feedstock utilization for milk and meat production with diminishing disadvantageous, alternative hydrogen sink pathways, e.g. lactate, succinate, or ethanol formation (Ungerfeld and Pitta 2024, Shinkai et al. 2024, O’Hara et al. 2025). We note that a similar “redox equilibrium switch” role can be envisaged for the planktonic rumen Protozoa. They have hydrogenosomes and endosymbiont methanogens, similar to those described in AGF. Hence, a similar regulation molecular machinery exists both in the liquid and fiber attached communities of the herbivores. A more exhaustive discussion of ruminal protozoa is out of scope of the present review.

### Harnessing AGF for biotechnological lignocellulose decomposition

**Figure 5.**
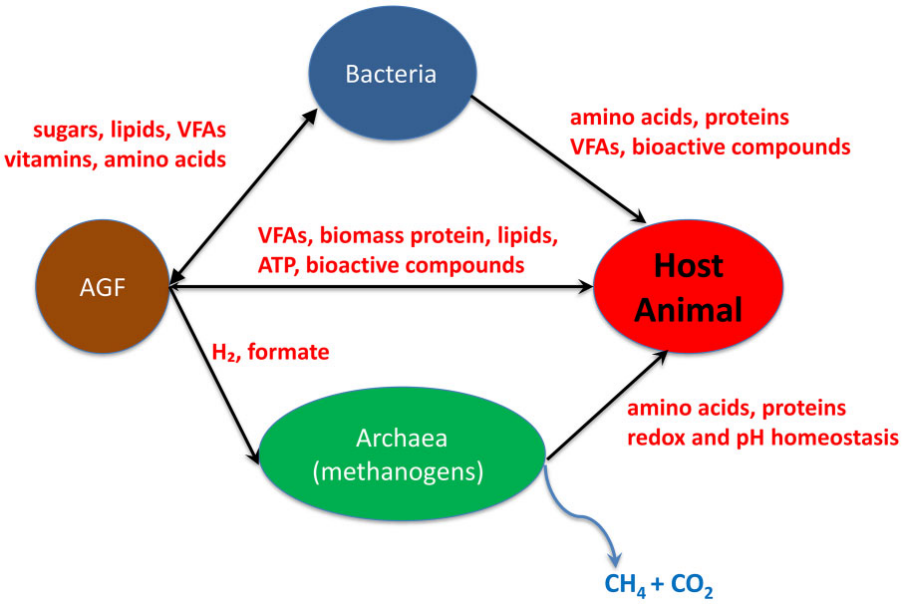
Schematic metabolic interactions in rumen microbial ecology. VFA = volatile fatty acids. Methanogenic archaea produce CH_4_ + CO_2_, which is an energetic loss and greenhouse gas unless it is utilized as renewable energy carrier.

There are biochemical and microbiological pathways to fully recover the chemical energy available after lignocellulose degradation. Rationally designed microbial ecosystems, comprising pure cultures of strains participating in the cofermentation are particularly useful in exploitation of the multifarious process of lignocellulose degradation and utilization (Fig. 5). This sensible principle is verified/tested in the next examples.

Henske et al. (2018) demonstrated that *Neocallimastix californiae* and *Anaeromyces robustus* efficiently converted lignocellulose to free glucose in batch cultures and the released substrate sugars were readily utilized by *Saccharomyces cerevisiae* for ethanol production (Henske et al. 2018). In a similar set of experiments, a bipartisan community successfully cooperated in a two-stage conversion of biomass to ethanol employing a coculture of the AGF *P. ruminantium* strain C1A and *Escherichia coli* strain K011 (Ranganathan et al. 2017).

High dietary fiber concentration facilitated the proliferation of AGF and members of phylum *Bacillota* (formerly *Firmicutes*), including the families *Ruminococcaceae* and *Succiniclasticum* (Plaizier et al. 2008, Boots et al. 2013, Kumar and Chandra 2020, Tapio et al. 2017, Fliegerova et al. 2021). AGF may also play a role in releasing lignocellulose-bound proteins (Lee et al. 2000, Gordon and Phillips 1998, Hartinger et al. 2018).

Cocultures of methanogens (M) with either AGF or bacteria (B) demonstrated elevated degradation of rice and wheat straw total fiber dry matter (Lee et al. 2000, Paul et al. 2004, Hartinger et al. 2019) relative to the M+B system. In a similar study, enrichments from rumen content were compared to evaluate their respective ability to degrade lignocellulose and produce CH_4_ (132). CO_2_ reduction to CH_4_ is an attractive H_2_ sink, which combines lignocellulose utilization to renewable energy (CH_4_) production (Ma et al. 2020, Edwards et al. 2017, Urrutia and Haryatine 2017, Mobashar et al. 2019).

Some AGF cannot be isolated in axenic cultures, they can sustain their life only together with their preferred symbiont methanogen partner (Mountfort and Orpin 1994, Bernalier et al. 1992, Swift et al. 2021). The AGF+M interaction was established (Burk and Van Dien 2016, Orpin and Joblin 1997) offering substantial biotechnological importance in the microbiological ecosystems.

Coculture metatranscriptomics demonstrated the upregulation of 105 genes encoding CAZymes representing 12% of total predicted CAZymes in the AGF *A. robustus*, when it thrived in a syntrophic coculture with the methanogen *Methanobacterium bryantii* (Swift et al. 2019) in an interesting microbial ecology interkingdom relationship.

Procházka et al. (2012) tested AGF isolates for their ability to integrate into the biogas producing anaerobic ecosystem. Batch cultures, fed batch cultures, and semicontinuous reactors, fed with anaerobic sludge from pig slurry and various kinds of lignocellulosic substrates (celluloses, maize, and grass silage), were inoculated with various AGF strains. All experiments showed positive effect of AGF on the biogas yield and quality (Procházka et al. 2012). Nevertheless, all cultivation experiments indicated that AGF could not survive for long time. The positive augmentation effect was observed, but apparently the inoculated fungi found themselves in an unfavorable bacterial environment and gradually ceased to function. Dollhofer et al. (2018) reported that pretreatment of hay solids with *Neocallimastix frontalis* isolates accelerated biomass degradation and improved biogas production in batch cultures, despite inhibitory volatile fatty acid (VFA) accumulation. The activity and viability of *N. frontalis* isolates decreased in time, which suggested that long term exploitation of AGF in biogas reactors might be a challenge in this microbial ecology.

In a two-stage biogas reactor system the contribution of *Piromyces rhizinflata* YM600 to H_2_, CH_4_, and VFA generation was studied (Nkemka et al. 2015). The initial production rates were elevated relative to the controls, but the final product yields were not improved.

Akin et al. (1990) tested two monocentric (Piromyces and Neocallimastix) and three polycentric (unnamed polycentric and Orpinomyces) AGF for decomposition of leaf blades and stems of Bermuda grass. All isolates degraded essentially the same amount of the leaf dry matter (∼70%,) in 9 days, although the digestion rate varied.

The dissimilar predominance of potential bacterial partners, i.e. the phylum *Bacillota* (formerly *Firmicutes*) in the biogas reactor versus phylum *Bacteroidetes* in the rumen, may be responsible for the distinct interaction mechanisms between the fungal and bacterial partners in the various microbial environments (Liang et al. 2023, Tapio et al. 2017, Fliegerova et al. 2021).

Coexistence between anaerobic fungi (AF) and bacteria can be antagonistic (Trinci et al. 1994), and the situation is complicated by the fact that bacteria outnumber fungi in the rumen by several orders of magnitude (Bernalier et al. 1992). In cocultures of AGF *A. robustus* or *Caecomyes churrovis* and rumen bacterium *Fibrobacter succinogenes* strain UWB7 fungal growth was negatively influenced (Swift et al. 2019). In this system, AGF and the cellulolytic bacterium competed for the same substrate, hence the antagonistic interaction response may not be very surprising. Nonetheless, the cellulolytic bacteria take advantage of the penetration and disruption of plant tissue by the fungal rhizoids (Lee et al. 2000).

The growth, activity, and homeostatic regulation of AGF containing microbial ecosystems depend mainly on the substrate availability and specificity of partner microbes (Henderson et al. 2015). Future studies are needed to fully understand the drivers and mechanisms of the communication and metabolite exchange networks within the complex microbial ecologies (Bhagat et al. 2023).

### Enzyme production

Ruminal AGF and their bacterial partners greatly contribute to lignocellulosic biomass degradation in the animal host (Mountfort and Asher 1985, Pearce and Bauchop 1985, Williams and Orpin 1987, Wood et al. 1986, Borneman et al. 1991). The majority of the gut fungal enzymatic activities are extracellular or confined to the membrane fraction (Williams and Orpin 1987, Hebraud and Fevre 1990, Li and Calza 1991, Tenuissen et al. 1992, Garcia-Campayo and Wood 1993, Vardakou et al. 2008, Wang et al. 2014, Lima and de Lucas 2022).

Dagar et al. (2018) evaluated sixteen strains of monocentric and polycentric AF for cellulase, xylanase, and esterase activities. *Neocallimastix* and *Orpinomyces* strains exhibited the highest lignocellulolytic activities.

Several enzymes from AGF have been successfully expressed heterologously in bacteria and yeast (Butkovich et al. 2025, Haitjema et al. 2014, Lankiewicz et al. 2022, Wang et al. 2014, Lima and de Lucas 2022, Dagar et al. 2018, Xue et al. 1992, Gilbert et al. 1992, Lee et al. 1993, Liu et al. 1999, Li et al. 2004, Cheng et al. 2014, O’Malley et al. 2012, Morrison et al. 2016, Xue et al. 1992). These studies indicate significant potential for both basic and applied research of AGF lignocellulolytic enzymes, which are currently still waiting for exploitation in various biotechnological utilizations.

In an attempt to overcome the hurdles associated with large-scale production of the exoenzyme producer AGF, the AF supernatants were also positively tested as silage pretreatment agents (Hartinger et al. 2022), recently.

Solomon et al. (2016) reported that secreted fungal enzymes of AGF strains readily hydrolyzed cellobiose, filter paper, avicel, and reed canary grass substrates. Members of the phylum Neocallimastigomycota, (genus *Piromyces* in particular) showed a 300% increase in xylan-degradation activity when compared with commercial *Aspergillus* enzymes. AGF are also rich in hemicellulases (notably GH10) and polysaccharide deacetylases (Lionetti et al. 2010). Pectin decomposition was supported by GH88s, GE, and PL (Bundhoo et al. 2013).

## Future outlook

The food and feed processing biotechnology sector as well as industrial production of platform chemicals and renewable energy carriers increasingly rely on lignocellulosic raw materials as we progress towards sustainable circular economy (Wirth et al. 2012, Boots et al. 2013, Harms et al. 2011, Chandra and Yadav 2021, Keasling et al. 2021, Ejaz et al. 2021, Kuhad et al. 2011, Nargotra et al. 2022). Despite their confirmed outstanding capabilities, anaerobic eukaryotes have been largely overlooked and underutilized in this context (Haitjema et al. 2017, Gilmore et al. 2015, Hsin et al. 2025, Simpson et al. 2008, Hakulinen et al. 2013, Hooker et al. 2023). AGF are usually present in low numerical abundance in the various anaerobic microbial ecosystems but they have disproportionally high biological activities. They play significant role in the degradation of ingested plant cellulosic fibers in herbivores by both invasive rhizoidal growth and by producing a vast array of polysaccharide-degrading enzymes (Orpin 1977b, Chen et al. 2024). AGF improve productivity and contribute to greenhouse gas emission control in the animal husbandries. Current studies focus primarily on the functional activities of members of bacteria kingdom and ignore contribution of other constituents of the anaerobic microbial world, including unicellular eukaryotes, such as AGF. In future research efforts a holistic “omics” approach can take us to a thorough understanding and management of the complex microbial ecology networks (Fig. 5).

The challenges in maintaining consistent laboratory cultures hindered the exploration of AGF for numerous industrial applications. Advancements in escalating and increasingly affordable molecular analytical techniques greatly aided the study of complex microbial ecosystems. Understanding the physiology and biotechnological capabilities of these microorganisms elevate them into the limelight of anaerobic lignocellulose processing technologies (Meili et al. 2023, Hooker et al. 2023). AGF enzyme cocktails have exhibited outstanding efficiency (Hooker et al. 2023).

The interkingdom symbiotic collaborations should be exploited more vigorously in future research to uncover networking between eukaryotic AGF and archaeal and bacterial partners in the mutually beneficial conversion of lignocellulosic biomass to commodity manufacturing, e.g. organic acids, lipids, and amino acids (Hooker et al. 2019). This complies with the concept of circular economy.

Lignocellulosic biomass will keep growing in importance as a cost-effective, widely available nonedible feedstock for second-generation biofuels, such as hydrogen, methane, and bioethanol (Bhat 2000, Tirado-Acevedo et al. 2010). In this context, the regulation and management of the sensitive redox equilibria among and within the members of the mixed microbial ecosystems is needed for high yields and selective production. The unique hydrogenosome biochemical machinery of AGF and protozoa should emerge as underscored major players of the regenerative industrial economy model that strives to minimize waste and increase productivity (Mohan et al. 2019).

## References

[bib1] Abera GB, Bhusal A, Anderson TO et al. Mitigating ammonia inhibition in in-situ biomethanation using anaerobic moving bed biofilm reactor. 2025;13:1189355. 10.1016/j.jece.2025.118355.

[bib2] Abramson M, Shoseyov O, Shani Z. Plant cell wall reconstruction toward improved lignocellulosic production and processability. Plant Sci. 2010;178:61–72. 10.1016/j.plantsci.2009.11.003.

[bib3] Aguilar A, Wohlgemuth R, Twardowski T. Perspectives on bioeconomy. New Biotechnol. 2018;40:181–4. 10.1016/j.nbt.2017.06.012. 28711519

[bib4] Aguilera-Campos KI, Boisard J, Törnblom V. et al. Anaerobic breviate protist survival in microcosms depends on microbiome metabolic function. ISME J. 2025;171:wraf171. 10.1093/ismejo/wraf171. PMC12453579 40795332

[bib5] Akin DE, Borneman WS, Lyon CE. Degradation of leaf blades and stems by monocentric and polycentric isolates of ruminal fungi. Anim Feed Sci Tech. 1990;31:205–21. 10.1016/0377-8401(90)90125-R.

[bib6] Akin DE, Borneman WS. Role of rumen fungi in fiber degradation. J Dairy Sci. 1990;73:3023–32. 10.3168/jds.S0022-0302(90)78989-8. 2178175

[bib7] Bainbridge ML, Saldinger LK, Barlow JW. et al. Alteration of rumen bacteria and protozoa through grazing regime as a tool to enhance the bioactive fatty acid content of bovine milk. Front Microbiol. 2018;9:904. 10.3389/fmicb.2018.00904. 29867815 PMC5951984

[bib8] Bauchop T, Mountfort DO. Cellulose fermentation by a rumen anaerobic fungus in both the absence and the presence of rumen methanogens. Appl Environ Microbiol. 1981;42:1103–10. 10.1128/aem.42.6.1103-1110.1981. 16345902 PMC244160

[bib9] Beauchemin KA, Ungerfeld EM, Eckard RJ. et al. Fifty years of research on rumen methanogenesis: lessons learned and future challenges for mitigation. Animal. 2020;14:s2–s16. 10.1017/S1751731119003100. 32024560

[bib10] Bernalier A, Fonty G, Bonnemoy F. et al. Degradation and fermentation of cellulose by the rumen anaerobic fungi in axenic cultures or in association with cellulolytic bacteria. Curr Microbiol. 1992;25:143–8. 10.1007/BF01571022.

[bib11] Bhagat NR, Kumar S, Kumari R. et al. A review on rumen anaerobic fungi: current understanding on carbohydrate fermentation and roughages digestion in ruminants. Appl Biochem Microbiol. 2023;59:231–49. 10.1134/S0003683823030043.

[bib12] Bhat MK. Cellulases and related enzymes in biotechnology. Biotechnol Adv. 2000;18:355–83. 10.1016/s0734-9750(00)00041-0. 14538100

[bib13] Billings AF, Fortney JL, Hazen TC. et al. Genome sequence and description of the anaerobic lignin-degrading bacterium *Tolumonas lignolytica* sp. nov. Stand Genom Sci. 2015;10:106. 10.1186/s40793-015-0100-3. PMC4653933 26594307

[bib14] Bomble YJ, Lin CY, Amore A. et al. Lignocellulose deconstruction in the biosphere. Curr Opin Chem Biol. 2017;41:61–70. 10.1016/j.cbpa.2017.10.013. 29100023

[bib15] Boots B, Lillis L, Clipson N. et al. Responses of anaerobic rumen fungal diversity (phylum Neocallimastigomycota) to changes in bovine diet. J Appl Microbiol. 2013;114:626–35. 10.1111/jam.12067. 23163953

[bib16] Borneman WS, Ljungdahl LG, Hartley RD. et al. Isolation and characterization of p-coumaroyl esterase from the anaerobic fungus *Neocallimastix* strain MC-2. Appl Environ Microbiol. 1991;57:2337–44. 10.1128/aem.57.8.2337-2344.1991. 1768103 PMC183573

[bib17] Brown JL, Perisin MA, Swift CL. et al. Co-cultivation of anaerobic fungi with *Clostridium acetobutylicum* bolsters butyrate and butanol production from cellulose and lignocellulose. J Industrial Microbiol Biotechnol. 2022;49:kuac024. 10.1093/jimb/kuac024. PMC9923384 36367297

[bib18] Brown JL, Swift CL, Mondo SJ. et al. Co–cultivation of the anaerobic fungus *Caecomyces churrovis* with *Methanobacterium bryantii* enhances transcription of carbohydrate binding modules, dockerins, and pyruvate formate lyases on specific substrates. Biotechnol Biofuels. 2021;14:234. 10.1186/s13068-021-02083-w. 34893091 PMC8665504

[bib19] Bundhoo ZMA, Mudhoo A, Mohee R. Promising unconventional pretreatments for lignocellulosic biomass. Crit Rev Environ Sci Technol. 2013;43:2140–211. 10.1080/10643389.2012.672070.

[bib20] Burk MJ, Van Dien S. Biotechnology for chemical production: challenges and opportunities. Trends Biotechnol. 2016;34:187–90. 10.1016/j.tibtech.2015.10.007. 26683567

[bib21] Butkovich LV, Leggieri PA, Lillington SP et al. Separation of life stages within anaerobic fungi (Neocallimastigomycota) highlights differences in global transcription and metabolism. Fungal Gen Biol. 2025;176:103958. 10.1016/j.fgb.2024.103958. 39746393

[bib22] Chandra MRGS, Yadav PS. Recent trends and future prospective of fungal cellulases for environmental management. Appl Microbiol Biochem. 2021;2:247–56. 10.1016/B978-0-12-821406-0.00023-0.

[bib23] Chen X, Zhang X, Sun C. et al. New insights into anaerobic digestion of lignocellulosic wastes towards carbon neutrality: a review of current advancement and future prospects. J Water Process Eng. 2024;58:106584. 10.1016/j.jwpe.2024.106584.

[bib24] Cheng Y-S, Chen C-C, Huang C-H. et al. Structural analysis of a glycoside hydrolase family 11 xylanase from *Neocallimastix patriciarum*: insights into the molecular basis of a thermophilic enzyme. J Biol Chem. 2014;289:11020–8. 10.1074/jbc.M114.550905. 24619408 PMC4036243

[bib25] Dagar SS, Kumar S, Mudgil P. et al. Comparative evaluation of lignocellulolytic activities of filamentous cultures of monocentric and polycentric anaerobic fungi. Anaerobe. 2018;50:76–9. 10.1016/j.anaerobe.2018.02.004. 29454109

[bib26] Davies DR, Theodorou MK, Lawrence MI. et al. Distribution of anaerobic fungi in the digestive tract of cattle and their survival in faeces. Microbiology. 1993;139:1395–400. 10.1099/00221287-139-6-1395. 8360630

[bib27] Dey A, Sehgal JP, Puniya AK. et al. Influence of an anaerobic fungal culture (*Orpinomyces* sp.) administration on growth rate, ruminal fermentation and nutrient digestion in calves. Asian Aust J Anim Sci. 2004;17:820–4. 10.5713/ajas.2004.820.

[bib28] Dollhofer V, Dandikas V, Dorn-In S. et al. Accelerated biogas production from lignocellulosic biomass after pre-treatment with *Neocallimastix frontalis*. Biores Technol. 2018;264:219–27. 10.1016/j.biortech.2018.05.068. 29807329

[bib29] Drake H, Ivarsson M, Heim C. et al. Fossilized anaerobic and possibly methanogenesis-fueling fungi identified deep within the Siljan impact structure, Sweden. Commun Earth Environ. 2021;2:34. 10.1038/s43247-021-00107-9.

[bib30] Drula E, Garron ML, Dogan S et al. The carbohydrate-active enzyme database: functions and literature. Nucleic Acids Res. 2022;50:D571–7. 10.1093/nar/gkab1045. 34850161 PMC8728194

[bib31] Duan J, Liang J, Wang Y. et al. Kraft lignin biodegradation by *Dysgonomonas* sp. WJDL-Y1, a new anaerobic bacterial strain isolated from sludge of a pulp and paper mill. J Microbiol Biotechnol. 2016;26:1765–73. 10.4014/jmb.1602.02014. 27381334

[bib32] Edwards JE, Forster RJ, Callaghan TM. et al. PCR and Omics based techniques to study the diversity, ecology and biology of anaerobic fungi: insights, challenges and opportunities. Front Microbiol. 2017;8:1657. 10.3389/fmicb.2017.01657. 28993761 PMC5622200

[bib33] Ejaz U, Sohail M, Ghanemi A. Cellulases: from bioactivity to a variety of industrial applications. Biomimetics. 2021;6:44. 10.3390/biomimetics6030044. 34287227 PMC8293267

[bib34] Ek M, Gellerstedt G, Henriksson G. (eds), Wood Chemistry and Wood Biotechnology. Vol. 1. Berlin: Walter de Gruyter, 2009. 10.1515/9783110213409.

[bib35] Festel G. Economic aspects of industrial biotechnology. In: Advances in Biochemical Engineering/Biotechnology. Berlin: Springer, 2018. 10.1007/10_2018_70 30046916

[bib36] Firkins JL, Yu Z, Park T. et al. Extending Burk Dehority’s perspectives on the role of ciliate protozoa in the rumen. Front Microbiol. 2020;11:123. 10.3389/fmicb.2020.00123. 32184759 PMC7058926

[bib37] Firkins JL, Yu Z. Ruminant nutrition symposium: how to use data on the rumen microbiome to improve our understanding of ruminant nutrition. J Anim Sci. 2015;93:1450–70. 10.2527/jas.2014-8754. 26020167

[bib38] Fliegerova KO, Podmirseg SM, Vinzelj J. et al. The effect of a high-grain diet on the rumen microbiome of goats with a special focus on anaerobic fungi. Microorganisms. 2021;9:157. 10.3390/microorganisms9010157. 33445538 PMC7827659

[bib39] Floudas D, Binder M, Riley R. et al. The Paleozoic origin of enzymatic lignin decomposition reconstructed from 31 fungal genomes. Science. 2012;336:1715–19., 10.1126/science.1221748. 22745431

[bib40] Garcia-Campayo V, Wood TM. Purification and characterisation of a beta-D-xylosidase from the anaerobic rumen fungus *Neocallimastix frontalis*. Carbohydr Res. 1993;242:229–45. 10.1016/0008-6215(93)80037-f. 8495441

[bib41] Gigli-Bisceglia N, Engelsdorf T, Hamann T. Plant cell wall integrity maintenance in model plants and crop species-relevant cell wall components and underlying guiding principles. Cell Mol Life Sci. 2020;77:2049–77. 10.1007/s00018-019-03388-8. 31781810 PMC7256069

[bib42] Gilbert HJ, Hazlewood GP, Laurie JI. et al. Homologous catalytic domains in a rumen fungal xylanase: evidence for gene duplication and prokaryotic origin. Mol Microbiol. 1992;6:2065–72. 10.1111/j.1365-2958.1992.tb01379.x. 1406248

[bib43] Gilmore SP, Henske JK, O’Malley MA. Driving biomass breakdown through engineered cellulosomes. Bioengineered. 2015;6:204–8. 10.1080/21655979.2015.1060379. 26068180 PMC4601266

[bib44] Gordon GLR, Phillips MW. The role of anaerobic gut fungi in ruminants. Nutr Res Rev. 1998;11:133–68. 10.1079/NRR19980009. 19087463

[bib45] Gruninger RJ, Puniya AK, Callaghan TM. et al. Anaerobic fungi (phylum Neocallimastigomycota): advances in understanding their taxonomy, life cycle, ecology, role and biotechnological potential. FEMS Microbiol Ecol. 2014;90:1–17. 10.1111/1574-6941.12383. 25046344

[bib46] Hagen LH, Brooke CG, Shaw CA. et al. Proteome specialization of anaerobic fungi during ruminal degradation of recalcitrant plant fiber. ISME J. 2021;15:421–34. 10.1038/s41396-020-00769-x. 32929206 PMC8026616

[bib47] Haitjema CH, Gilmore SP, Henske JK. et al. A parts list for fungal cellulosomes revealed by comparative genomics. Nat Microbiol. 2017;2:17087. 10.1038/nmicrobiol.2017.87. 28555641

[bib48] Haitjema CH, Solomon KV, Henske JK. et al. Anaerobic gut fungi: advances in isolation, culture, and cellulolytic enzyme discovery for biofuel production. Biotechnol Bioeng. 2014;111:1471–82. 10.1002/bit.25264. 24788404

[bib49] Hakulinen N, Gasparetti C, Kaljunen H. et al. The crystal structure of an extracellular catechol oxidase from the ascomycete fungus *Aspergillus oryzae*. J Biol Inorg Chem. 2013;18:917–29. 10.1007/s00775-013-1038-9. 24043469

[bib50] Hanafy RA, Dagar SS, Griffith GW. et al. Taxonomy of the anaerobic gut fungi (Neocallimastigomycota): a review of classification criteria and description of current taxa. Int J Syst Evol Micr. 2022;72:10.1099/ijsem.0.005322. 35776761

[bib51] Harms H, Schlosser D, Wick LY. Untapped potential: exploiting fungi in bioremediation of hazardous chemicals. Nat Rev Microbiol. 2011;9:177–92. 10.1038/nrmicro2519. 21297669

[bib52] Hartinger T, Edwards JE, Gómez Expósito R. et al. Differently pre-treated alfalfa silages affect the *in vitro* ruminal microbiota composition. Front Microbiol. 2019;10:2761. 10.3389/fmicb.2019.02761. 31849900 PMC6902091

[bib53] Hartinger T, Fliegerová K, Zebeli Q. Suitability of anaerobic fungi culture supernatant or mixed ruminal fluid as novel silage additives. Appl Microbiol Biotechnol. 2022;106:6819–32. 10.1007/s00253-022-12157-w. 36100752 PMC9529681

[bib54] Hartinger T, Gresner N, Südekum K-H. Does intra-ruminal nitrogen recycling waste valuable resources? A review of major players and their manipulation. J Anim Sci Biotechnol. 2018;9:33. 10.1186/s40104-018-0249-x. 29721317 PMC5911377

[bib55] Hartinger T, Zebeli Q. The present role and new potentials of anaerobic fungi in ruminant nutrition. J Fungi. 2021;7:200. 10.3390/jof7030200. PMC8000393 33802104

[bib56] Hebraud M, Fevre M. Purification and characterization of an extracellular beta-xylosidase from the rumen anaerobic fungus *Neocallimastix frontalis*. FEMS Microbiol Lett. 1990;60:11–6. 10.1111/j.1574-6968.1990.tb03853.x. 2126511

[bib57] Henderson G, Cox F, Ganesh S. et al. Rumen microbial community composition varies with diet and host, but a core microbiome is found across a wide geographical range. Sci Rep. 2015;5:14567. 10.1038/srep14567. 26449758 PMC4598811

[bib58] Henske JK, Gilmore SP, Knop D. et al. Transcriptomic characterization of *Caecomyces churrovis*: a novel, non-rhizoid-forming lignocellulolytic anaerobic fungus. Biotechnol Biofuels. 2017;10:305–. 10.1186/s13068-017-0997-4. 29270219 PMC5737911

[bib59] Henske JK, Wilken SE, Solomon KV. et al. Metabolic characterization of anaerobic fungi provides a path forward for bioprocessing of crude lignocellulose. Biotechnol Bioeng. 2018;115:874–84. 10.1002/bit.26515. 29240224

[bib60] Hess M, Paul SS, Puniya AK. et al. Anaerobic fungi: past, present, and future. Front Microbiol. 2020;11:584893. 10.3389/fmicb.2020.584893. 33193229 PMC7609409

[bib61] Himmel ME, Ding SY, Johnson DK. et al. Biomass recalcitrance: engineering plants and enzymes for biofuels production. Science. 2007;315:804–7. 10.1126/science.1137016. 17289988

[bib62] Hoogzaad JA, Lembachar Y, Bąkowska O. et al. Climate Change Mitigation through the Circular Economy—A report for the Scientific and Technical Advisory Panel (STAP), to the Global Environment Facility (GEF). Amsterdam: Global Environment Facility, 2020.

[bib63] Hooker CA, Hanafy R, Hillman ET. et al. A genetic engineering toolbox for the lignocellulolytic anaerobic gut fungus *Neocallimastix frontalis*. ACS Synth Biol. 2023;12:1034–45. 10.1021/acssynbio.2c00502. 36920337 PMC11677189

[bib64] Hooker CA, Lee KZ, Solomon KV. Leveraging anaerobic fungi for biotechnology. Curr Opinion Biotechnol. 2019;59:103–10. 10.1016/j.copbio.2019.03.013. 31005803

[bib65] Hsin K-T, Lee HT, Huang Y-C. et al. Lignocellulose degradation in bacteria and fungi: cellulosomes and industrial relevance. Front Microbiol. 2025;16:1583746. 10.3389/fmicb.2025.1583746. 40351319 PMC12063362

[bib66] Hu Y, Bao Y, Meng J et al. Biodegradation sequence of coal organic matter and mechanism of biomethane formation in secondary biogenic gas accumulation areas. Org Geochem. 2025;205:104996. 10.1016/j.orggeochem.2025.104996.

[bib67] Janssen PH, Kirs M. Structure of the archaeal community of the rumen. Appl Env Microbiol. 2008;74:3619–25. 10.1128/AEM.02812-07. 18424540 PMC2446570

[bib68] Janusz G, Pawlik A, Sulej J. et al. Lignin degradation: microorganisms, enzymes involved, genomes analysis and evolution. FEMS Microbiol Rev. 2017;41:941–62. 10.1093/femsre/fux049. 29088355 PMC5812493

[bib69] Jones AL, Pratt CJ, Meili CH. et al. Anaerobic gut fungal communities in marsupial hosts. mBio. 2024;15:e03370–23. 10.1128/mbio.03370-23. 38259066 PMC10865811

[bib70] Kakuk B, Wirth R, Maróti G. et al. Early response of methanogenic archaea to H_2_ as evaluated by metagenomics and metatranscriptomics. Microb Cell Fact. 2021;20:127. 10.1186/s12934-021-01618-y. 34217274 PMC8254922

[bib71] Kazda M, Langer S, Bengelsdorf FR. Fungi open new possibilities for anaerobic fermentation of organic residues. Energy Sust Soc. 2014;4:6. 10.1186/2192-0567-4-6.

[bib72] Keasling J, Martin HG, Lee TS. et al. Microbial production of advanced biofuels. Nat Rev Microbiol. 2021;19:701–15. 10.1038/s41579-021-00577-w. 34172951

[bib73] Kim M, Morrison M, Yu Z. Phylogenetic diversity of bacterial communities in bovine rumen as affected by diets and microenvironments. Folia Microbiol. 2011;56:453–8. 10.1007/s12223-011-0066-5. 21901294

[bib74] Kovács E, Szűcs C, Farkas A. et al. Pretreatment of lignocellulosic biogas substrates by filamentous fungi. J Biotechnol. 2022;360:160–70. 10.1016/j.jbiotec.2022.10.013. 36273669

[bib75] Kuhad RC, Gupta R, Singh A. Microbial cellulases and their industrial applications. Enzyme Res. 2011;2011:1. 10.4061/2011/280696. PMC3168787 21912738

[bib76] Kumar A, Chandra R. Ligninolytic enzymes and its mechanisms for degradation of lignocellulosic waste in environment. Heliyon. 2020;6:e03170. 10.1016/j.heliyon.2020.e03170vvvvvv. 32095645 PMC7033530

[bib77] Lankiewicz TS, Lillington SP, O’Malley MA. Enzyme discovery in anaerobic fungi (Neocallimastigomycetes) enables lignocellulosic biorefinery innovation. Microbiol Mol Biol Rev. 2022;86:e0004122. 10.1128/mmbr.00041-22. 35852448 PMC9769567

[bib78] Laundon D, Chrismas N, Wheeler G. et al. Chytrid rhizoid morphogenesis resembles hyphal development in multicellular fungi and is adaptive to resource availability. Proc Royal Soc B. 2020;287:20200433. 10.1098/rspb.2020.0433. PMC7341943 32517626

[bib79] Lebo SE Jr, Gargulak JD, McNally TJ. Lignin. Kirk-Othmer Encyclopedia of Chemical Technology. Hoboken: John Wiley & Sons, Inc, 2001. 10.1002/0471238961.12090714120914.a01.pub2. ISBN 978-0-471-23896-6

[bib80] Lee C, Beauchemin KA. A review of feeding supplementary nitrate to ruminant animals: nitrate toxicity, methane emissions. and production performance. Can J Anim Sci. 2014;84:557–70. 10.4141/CJAS-2014-069.

[bib81] Lee JMT, Hu Y, Zhu H. et al. Cloning of a xylanase gene from the ruminal fungus *Neocallimastix patriciarum* 27 and its expression in *Escherichia coli*. Can J Microbiol. 1993;39:134–9. 10.1139/m93-020. 8439870

[bib82] Lee SS, Ha JK, Cheng K-J. Relative contributions of bacteria, protozoa, and fungi to *in vitro* degradation of orchard grass cell walls and their interactions. Appl Environ Microbiol. 2000;66:3807–13. 10.1128/AEM.66.9.3807-3813.2000. 10966394 PMC92224

[bib83] Lewis WH, Sendra KM, Embley TM. et al. Morphology and phylogeny of a new species of anaerobic ciliate, *Trimyema finlayin*. sp., with endosymbiotic methanogens. Front Microbiol. 2018;9:140. 10.3389/fmicb.2018.00140. 29515525 PMC5826066

[bib84] Li X, Calza RE. Purification and characterization of an extracellular β-glucosidase from the rumen fungus *Neocallimastix frontalis* EB188. Enzyme Microbiol Technol. 1991;13:622–528. 10.1016/0141-0229(91)90075-L.

[bib85] Li X-L, Ljungdahl LG, Ximenes EA. et al. Properties of a recombinant β-glucosidase from polycentric anaerobic fungus *Orpinomyces* PC-2 and its application for cellulose hydrolysis. Appl Biochem Biotechnol. 2004;113–16:233–50. 10.1385/abab:113:1-3:233. PMC5890932 15054209

[bib86] Li Y, Li Y, Jin W. et al. Combined genomic, transcriptomic, proteomic, and physiological characterization of the growth of *Pecoramyces* sp. F1 in monoculture and co-culture with a syntrophic methanogen. Front Microbiol. 2019;10:435. 10.3389/fmicb.2019.00435. 30894845 PMC6414434

[bib87] Liang Z, Zhang J, Ahmad AA. et al. Forage lignocellulose is an important factor in driving the seasonal dynamics of rumen anaerobic fungi in grazing yak and cattle. Microbiol Spectr. 2023;11:e00788–23. 10.1128/spectrum.00788-23. 37707448 PMC10581131

[bib88] Liggenstoffer AS, Youssef NH, Couger MB. et al. Phylogenetic diversity and community structure of anaerobic gut fungi (phylum Neocallimastigomycota) in ruminant and non-ruminant herbivores. ISME J. 2010;4:1225–35. 10.1038/ismej.2010.49. 20410935

[bib89] Lillington SP, Leggieri PA, Heom KA. et al. Nature’s recyclers: anaerobic microbial communities drive crude biomass deconstruction. Curr Opinion Biotechnol. 2020;62:38–47. 10.1016/j.copbio.2019.08.015. 31593910

[bib90] Lima MS, de Lucas RC. Co-cultivation, co-culture, mixed culture, and microbial consortium of fungi: an understudies strategy for biomass conversion. Front Microbiol. 2022;12:837685. 10.3389/fmicb.2021.837685. 35126339 PMC8811191

[bib92] Lionetti V, Francocci F, Ferrari S. et al. Engineering the cell wall by reducing de-methyl-esterified homogalacturonan improves saccharification of plant tissues for bioconversion. Proc Natl Acad Sci USA. 2010;107: 616–21. 10.1073/pnas.090754910. 20080727 PMC2818903

[bib93] Liu J-H, Selinger BL, Tsai C-F. et al. Characterization of a *Neocallimastix patriciarum* xylanase gene and its product. Can J Microbiol. 1999;45:970–4. 10.1139/w99-092. 10588045

[bib94] Lockhart RJ, Van Dyke MI, Beadle IR. et al. Molecular biological detection of anaerobic gut fungi (Neocallimastigales) from landfill sites. Appl Environ Microbiol. 2006;72:5659–61. 10.1128/AEM.01057-06. 16885325 PMC1538735

[bib95] López-García A, Saborío-Montero A, Gutiérrez-Rivas M. et al. Fungal and ciliate protozoa are the main rumen microbes associated with methane emissions in dairy cattle. Gigascience. 2022;11:giab088. 10.1093/gigascience/giab088. 35077540 PMC8848325

[bib96] Ma J, Zhong P, Li Y. et al. Hydrogenosome, pairing anaerobic fungi and H_2_-utilizing microorganisms based on metabolic ties to facilitate biomass utilization. Fungi. 2022;8:338. 10.3390/jof8040338. PMC9026988 35448569

[bib97] Ma Y, Li Y, Li Y. et al. The enrichment of anaerobic fungi and methanogens showed higher lignocellulose degrading and methane producing ability than that of bacteria and methanogens. World J Microbiol Biotechnol. 2020;36:125. 10.1007/s11274-020-02894-3. 32712756

[bib98] Martin C, Williams AG, Michalet-Doreau B. Isolation and characteristics of the protozoal and bacterial fractions from bovine ruminal contents. J Anim Sci. 1994;72:2962–8. 10.2527/1994.72112962x. 7730192

[bib99] McMahon S, Parnell J. Weighing the deep continental biosphere. FEMS Microbiol Ecol. 2014;87:113–20. 10.1111/1574-6941.12196. 23991863

[bib100] Meili CH, Jones AL, Arreola AX. et al. Patterns and determinants of the global herbivorous mycobiome. Nat Comm. 2023;14:3798. 10.1038/s41467-023-39508-z. PMC10293281 37365172

[bib101] Meili CH, TagElDein MA, Jones AL. et al. Diversity and community structure of anaerobic gut fungi in the rumen of wild and domesticated herbivores. Appl Environ Microbiol. 2024;90:e0149223. 10.1128/aem.01492-23. 38299813 PMC10880628

[bib102] Mobashar M, Hummel J, Blank R. et al. Contribution of different rumen microbial groups to gas, short-chain fatty acid and ammonium production from different diets-an approach in an in vitro fermentation system. J Anim Physiol Anim Nutr. 2019;103:17–28. 10.1111/jpn.12996. 30280429

[bib103] Mohan SV, Dahiya S, Amulya K. et al. Can circular bioeconomy be fueled by waste biorefineries—a closer look. Biores Technol Rep. 2019;7:100277. 10.1016/j.biteb.2019.100277.

[bib104] Mondo SJ, He G, Sharma A. et al. Consecutive low-frequency shifts in a/T content denote nucleosome positions across microeukaryotes. iScience. 2025;28:112472. 10.1016/j.isci.2025.112472. 40491964 PMC12146615

[bib105] Moreira EA, Persinoti GF, Menezes LR et al. Complementary contribution of fungi and bacteria to lignocellulose digestion in the food stored by a neotropical higher termite. Front Ecol Evol. 2021;9:632590. 10.3389/fevo.2021.632590.

[bib106] Morrison JM, Elshahed MS, Youssef NH. Defined enzyme cocktail from the anaerobic fungus *Orpinomyces* sp. strain C1A effectively releases sugars from pretreated corn stover and switchgrass. Sci Rep. 2016;6:29217. 10.1038/srep29217. 27381262 PMC4933900

[bib107] Mountfort DO, Orpin CG. (eds), Anaerobic Fungi: Biology, Ecology, and Function. New York: Marcel Dekker, 1994, 290. ISBN: 9780824789480.

[bib108] Mountfort DO, Asher RA. Production and regulation of cellulase by two strains of the rumen anaerobic fungus *Neocallimastix frontalis*. Appl Environ Microbiol. 1985;49: 1314–22. 10.1128/aem.49.5.1314-1322.1985. 3923931 PMC238548

[bib109] Mura E, Edwards J, Kittelmann S. et al. Anaerobic fungal communities differ along the horse digestive tract. Fung Biol. 2019;123:240–46. 10.1016/j.funbio.2018.12.004. 30798879

[bib110] Nargotra P, Sharma V, Lee YC. et al. Microbial lignocellulolytic enzymes for the effective valorization of lignocellulosic biomass: a review. Catalysts. 2022;13:83. 10.3390/catal13010083.

[bib111] Newbold CJ, De La Fuente G, Belanche A. et al. The role of ciliate protozoa in the rumen. Front Microbiol. 2015;6:1313. 10.3389/fmicb.2015.01313. PMC4659874 26635774

[bib113] Nicholson MJ, Theodorou MK, Brookman JL. Molecular analysis of the anaerobic rumen fungus *Orpinomyces*—insights into an AT-rich genome. Microbiology. 2005;151:121–33. 10.1099/mic.0.27353-0. 15632432

[bib114] Nkemka VN, Gilroyed B, Yanke J. et al. Bioaugmentation with an anaerobic fungus in a two-stage process for biohydrogen and biogas production using corn silage and cattail. Biores Technol. 2015;185:79–88. 10.1016/j.biortech.2015.02.100. 25755016

[bib115] O’Hara E, Chomistek N, Terry SA. et al. Assessing the impact of the methane inhibitors 3-Nitrooxypropanol (3-NOP) and canola oil on the rumen anaerobic fungi. Animals. 2025;15:1230. 10.3390/ani15091230. 40362045 PMC12071074

[bib116] O’Malley MA, Theodorou MK, Kaiser CA. Evaluating expression and catalytic activity of anaerobic fungal fibrolytic enzymes cative to *Piromyces* sp. E2 in *Saccharomyces cerevisiae*. Environ Prog Sustain Energy. 2012;31:37–46. 10.1002/ep.10614.

[bib117] Orpin CG, Joblin KN. The rumen anaerobic fungi. In: Hobson P.N., Stewart C.S. (eds), The Rumen Microbial Ecosystem. Amsterdam: Springer, 1997, 140–95. ISBN: 978-0-7514-0366-4 1997.

[bib118] Orpin CG. Studies on the rumen flagellate *Neocallimastix frontalis*. J Gen Microbiol. 1975;91:249–62. 10.1099/00221287-91-2-249. 1462

[bib119] Orpin CG. The rumen flagellate *Piromonas communis*: its life-history and invasion of plant material in the rumen. J Gen Microbiol. 1977a;99:107–17. 10.1099/00221287-99-1-107. 16983

[bib120] Orpin CG. The occurrence of chitin in the cell walls of the rumen organisms *Neocallimastix frontalis, Piromonas communis* and *Sphaeromonas communis*. J Gen Microbiol. 1977b;99:215–8. 10.1099/00221287-99-1-215. 864435

[bib121] Panahi HKS, Dehhaghi M, Guillemin GJ. et al. A comprehensive review on anaerobic fungi applications in biofuels production. Sci Total Environ. 2022;829:154521. 10.1016/j.scitotenv.2022.154521. 35292323

[bib122] Paul S, Kamra D, Sastry V. et al. Effect of administration of an anaerobic gut fungus isolated from wild blue bull (*Boselaphus tragocamelus*) to buffaloes (*Bubalus bubalis*) on *in vivo* ruminal fermentation and digestion of nutrients. Anim Feed Sci Technol. 2004;115:143–57. 10.1016/j.anifeedsci.2004.01.010.

[bib123] Pearce PD, Bauchop T. Glycosidases of the rumen anaerobic fungus *Neocallimastix frontalis* grown on cellulosic substrates. Appl Environ Microbiol. 1985;49:1265–9. 10.1128/aem.49.5.1265-1269.1985. 4004240 PMC238540

[bib124] Perez HG, Stevenson CK, Lourenco JM. et al. Understanding rumen microbiology: an overview. Encyclopedia. 2024;4:148–57. 10.3390/encyclopedia4010013.

[bib125] Picard KT. Coastal marine habitats harbor novel early-diverging fungal diversity. Fung Ecol. 2017;25:1–13. 10.1016/S0965-1748(97)00002-7.

[bib126] Plaizier JC, Krause DO, Gozho GN. et al. Subacute ruminal acidosis in dairy cows: the physiological causes, incidence and consequences. Veter J. 2008;176:21–31. 10.1016/j.tvjl.2007.12.016. 18329918

[bib127] Pollegioni L, Tonin F, Rosini E. Lignin-degrading enzymes. FEBS J. 2015;282:1190–213. 10.1111/febs.13224. 25649492

[bib128] Priya M, Haridas A, Manilal VB. Anaerobic protozoa and their growth in biomethanation systems. Biodegradation. 2008;19:179–85. 10.1007/s10532-007-9124-8. 17492357

[bib129] Procházka J, Mrázek J, Štrosová L. et al. Enhanced biogas yield from energy crops with rumen anaerobic fungi. Eng Life Sci. 2012;12:343–51. 10.1002/elsc.201100076.

[bib130] Ralph J, Lundquist K, Brunow G. et al. Lignins: natural polymers from oxidative coupling of 4-hydroxyphenyl-propanoids. Phytochem Rev. 2004;3:29–60. 10.1023/B:PHYT.0000047809.65444.a4.

[bib131] Ranganathan A, Smith OP, Youssef NH. et al. Utilizing anaerobic fungi for two-stage sugar extraction and biofuel production from lignocellulosic biomass. Front Microbiol. 2017;8:1–10. 10.3389/fmicb.2017.00635. 28443088 PMC5387070

[bib132] Rao J, Lv Z, Chen G et al. Hemicellulose: structure, chemical modification, and application. Prog Polym Sci. 2023;140:101675. 10.1016/j.progpolymsci.2023.101675.

[bib133] Resch MG, Donohoe BS, Baker JO. et al. Fungal cellulases and complexed cellulosomal enzymes exhibit synergistic mechanisms in cellulose deconstruction. Energy Environ Sci. 2013;6:1858–67. 10.1039/C3EE00019B.

[bib134] Rogers JN, Stokes B, Dunn J. et al. An assessment of the potential products and economic and environmental impacts resulting from a billion ton bioeconomy. Biofuels Bioprod Bioref. 2017;11:110–28. 10.1002/bbb.1728.

[bib135] Romero P, Huang R, Jiménez E. et al. Evaluating the effect of phenolic compounds as hydrogen acceptors when ruminal methanogenesis is inhibited in vitro–Part 2. Dairy goats. Animal. 2023;17:100789. 10.1016/j.animal.2023.100789. 37087998

[bib136] Rotterova J, Breusing C, Cepicka I et al. Population genomic insights into syntrophic symbioses between marine anaerobic ciliates and intracellular methanogens. bioRxiv. 2025. 10.1101/2025.07.30.667679.

[bib137] Rubin EM. Genomics of cellulosic biofuels. Nature. 2008;454:841–5. 10.1038/nature07190. 18704079

[bib138] Sanjorjo RA, Tseten T, Kang M-K. et al. In pursuit of understanding the rumen microbiome. Fermentation. 2023;9:114. 10.3390/fermentation9020114.

[bib139] Saye LM, Navaratna TA, Chong JP. et al. The anaerobic fungi: challenges and opportunities for industrial lignocellulosic biofuel production. Microorganisms. 2021;9:694. 10.3390/microorganisms9040694. 33801700 PMC8065543

[bib140] Scarlat N, Dallemand JF, Monforti-Ferrario F. et al. The role of biomass and bioenergy in a future bioeconomy: policies and facts. Environ Dev. 2015;15:3–34. 10.1016/j.envdev.2015.03.006.

[bib141] Shinkai T, Takizawa S, Fujimori M et al. The role of rumen microbiota in enteric methane mitigation for sustainable ruminant production. 2024;37:360–9. 10.5713/ab.23.0301. PMC10838666 37946422

[bib142] Silva JP, Ticona ARP, Hamann PRV. et al. Deconstruction of lignin: from enzymes to microorganisms. Molecules. 2021;26:2299. 10.3390/molecules26082299. 33921125 PMC8071518

[bib143] Simpson SD, Forster RLS, Tran PL. et al. Bacteria and methods of use thereof. US Patent No. 12742149. 2008.

[bib144] Slaytor M, Veivers PC, Lo N. Aerobic and anaerobic metabolism in the higher termite *Nasutitermes walkeri* (Hill). Insect Biochem Mol Biol. 1997;27:291–303. 10.1016/S0965-1748(97)00002-7.

[bib145] Solomon KV, Haitjema CH, Henske JK. et al. Early-branching gut fungi possess a large, comprehensive array of biomass-degrading enzymes. Science. 2016;351:1192–5. 10.1126/science.aad1431. 26912365 PMC5098331

[bib146] Solomon R, Rute LMB, Kumar R. et al. Systematic discovery of bacterial symbionts in rumen ciliate protozoa. bioRxiv. 2025. 10.1101/2025.07.29.667580.

[bib147] Stabel M, Schweitzer T, Haack K. et al. Isolation and biochemical characterization of six anaerobic fungal strains from zoo animal feces. Microorganisms. 2021;9:1655. 10.3390/microorganisms9081655. 34442734 PMC8399178

[bib148] Sun Z, Liu Q, Li Y. et al. Deciphering the impact of lignin on anaerobic digestion: focus on inhibition mechanisms and methods for alleviating inhibition. ACS Omega. 2024;9:44033–41. 10.1021/acsomega.4c04375. 39524670 PMC11541797

[bib149] Suzuki H, MacDonald J, Syed K. et al. Comparative genomics of the white-rot fungi, *Phanerochaete carnosa* and *P. chrysosporium*, to elucidate the genetic basis of the distinct wood types they colonize. BMC Genomics. 2012;13:444. 10.1186/1471-2164-13-444. 22937793 PMC3463431

[bib150] Swift CL, Brown JL, Seppälä S. et al. Co-cultivation of the anaerobic fungus *Anaeromyces robustus* with *Methanobacterium bryantii* enhances transcription of carbohydrate active enzymes. J Ind Microbiol Biotechnol. 2019;6: 1427–33. 10.1007/s10295-019-02188-0. 31089985

[bib151] Swift CL, Louie KB, Bowen BP. et al. Cocultivation of anaerobic fungi with rumen bacteria establishes an antagonistic relationship. mBio. 2021;12:e0144221. 10.1128/mBio.01442-21. 34399620 PMC8406330

[bib152] Tapio I, Fischer D, Blasco L. et al. Taxon abundance, diversity, co-occurrence and network analysis of the ruminal microbiota in response to dietary changes in dairy cows. PLoS One. 2017;12:e0180260. 10.1371/journal.pone.0180260. eCollection. 28704445 PMC5509137

[bib153] Taxis TM, Wolff S, Gregg SJ. et al. The players may change but the game remains: network analyses of ruminal microbiomes suggest taxonomic differences mask functional similarity. Nucleic Acids Res. 2015;43:9600–12. 10.1093/nar/gkv973. 26420832 PMC4787786

[bib154] Teunissen MJ, Lahaye DHTP, Huis Veld JHJ. et al. Purification and characterization of an extracellular beta-glucosidase from the anaerobic fungus *Piromyces* sp. strain E2. Arch Microbiol. 1992;158:276–81. 10.1007/BF00245245.

[bib155] Theodorou MK, Mennim G, Davies DR. et al. Anaerobic fungi in the digestive tract of mammalian herbivores and their potential for exploitation. Proc Nutr Soc. 1996;55:913–26. 10.1079/pns19960088. 9004333

[bib156] Thongbunrod N, Chaiprasert P. Efficient methane production from agro-industrial residues using anaerobic fungal-rich consortia. World J Microbiol Biotechnol. 2024;40:239. 10.1007/s11274-024-04050-7. 38862848

[bib157] Tirado-Acevedo O, Chinn MS, Grunden AM. Production of biofuels from synthesis gas using microbial catalysts. Adv Appl Microbiol. 2010;70:57–92. 10.1016/S0065-2164(10)70002-2. 20359454

[bib158] Trinci APJ, Davies DR, Gull K. et al. Anaerobic fungi in herbivorous animals. Mycol Res. 1994;98: 129–52. 10.1016/S0953-7562(09)80178-0.

[bib159] Tripathi VK, Sehgal JP, Puniya AK. et al. Effect of administration of anaerobic fungi isolated from cattle and wild blue bull (*Boselaphus tragocamelus*) on growth rate and fibre utilization in buffalo calves. Arch Anim Nutr. 2007;61:416–23. 10.1080/17450390701556759. 18030922

[bib160] Ungerfeld EM, Pitta D. Review: biological consequences of the inhibition of rumen methanogenesis. Animal. 2024;1001170. 10.1016/j.animal.2024.101170. 38772773

[bib161] Urrutia NL, Harvatine KJ. Acetate dose-dependently stimulates milk fat synthesis in lactating dairy cows. J Nutr. 2017;147:763–9. 10.3945/jn.116.245001. 28331053

[bib162] Vardakou M, Dumon C, Murray JW. et al. Understanding the structural basis for substrate and inhibitor recognition in eukaryotic GH11 xylanases. J Mol Biol. 2008;375:1293–305. 10.1016/j.jmb.2007.11.007. 18078955

[bib163] Vizzarro A, Azim AA, Bassani I et al. Assessing the methanogenic activity of microbial communities enriched from a depleted reservoir. FEMS Microb Ecol. 2025;101:fiaf040. 10.1093/femsec/fia1040. PMC12054477 40234215

[bib164] Wang D, Zhao C, Liu S. et al. Effects of *Piromyces* sp. CN6 CGMCC 14449 on fermentation quality, nutrient composition and the in vitro degradation rate of whole crop maize silage. AMB Expr. 2019;9:1–8. 10.1186/s13568-019-0846-x. PMC6663944 31359220

[bib165] Wang H-C, Chen Y-C, Hseu R-S. Purification and characterization of a cellulolytic multienzyme complex produced by *Neocallimastix patriciarum* J11. Biochem Biophys Res Commun. 2014;451:190–5. 10.1016/j.bbrc.2014.07.088. 25073115

[bib166] Wang X, Liu X, Groenewald JZ. Phylogeny of anaerobic fungi (phylum Neocallimastigomycota), with contributions from yak in China. Antonie Van Leeuwenhoek. 2017;110:87–103. 10.1007/s10482-016-0779-1. 27734254 PMC5222902

[bib167] Wiedenhofer D, Pauliuk S, Mayer A. et al. Monitoring a sustainable circular economy: from the systems level to actors and organizations. In: Handbook of the Circular Economy. Cheltenham: Edward Elgar Publishing, 2020, 176–93. 10.4337/9781788972727.00022.

[bib168] Wilken SE, Monk JM, Leggieri PA. et al. Experimentally validated reconstruction and analysis of a genome-scale metabolic model of an anaerobic Neocallimastigomycota fungus. mSystems. 2021;6:e00002–21. 10.1128/msystems.00002. 33594000 PMC8561657

[bib169] Wilken SE, Seppälä S, Lankiewicz TS. et al. Genomic and proteomic biases inform metabolic engineering strategies for anaerobic fungi. Metabol Eng Commun. 2020;10:e00107. 10.1016/j.mec.2019.e00107. PMC6883316 31799118

[bib170] Williams AG, Orpin CG. Polysaccharide-degrading enzymes formed by three species of anaerobic rumen fungi grown on a range of carbohydrate substrates. Can J Microbiol. 1987;33:418–26. 10.1139/m87-071. 3607610

[bib171] Wirth R, Kovács E, Maróti G. et al. Characterization of a biogas-producing microbial community by short-read next generation DNA sequencing. Biotechnol Biofuels. 2012;5:41. 10.1186/1754-6834-5-41. 22673110 PMC3395570

[bib172] Wood TM, Wilson CA, McCrae SI. et al. A highly active extracellular cellulase from the anaerobic rumen fungus *Neocallimastix frontalis*. FEMS Microbiol Lett. 1986;34: 37–40. 10.1111/j.1574-6968.1986.tb01344.x.

[bib173] Xu Q, Qiao Q, Gao Y. et al. Gut microbiota and their role in health and metabolic disease of dairy cow. Front Nutr. 2021;8:701511. 10.3389/fnut.2021.701511. 34422882 PMC8371392

[bib174] Xue D, Liu T, Chen H. et al. Fungi are more sensitive than bacteria to drainage in the peatlands of the Zoige Plateau. Ecol Indic. 2021;124:107367. 10.1016/j.ecolind.2021.107367.

[bib175] Xue GP, Gobius KS, Orpin CG. A novel polysaccharide hydrolase cDNA (celD) from *Neocallimastix patriciarum* encoding three multi-functional catalytic domains with high endoglucanase, cellobiohydrolase and xylanase activities. J Gen Microbiol. 1992;138:2397–403. 10.1099/00221287-138-11-2397. 1479358

[bib176] Young LY, Frazer AC. The fate of lignin and lignin-derived compounds in anaerobic environments. Geomicrobiol J. 1987;5:261–93. 10.1080/01490458709385973.

[bib177] Youssef NH, Couger MB, Struchtemeyer CG. et al. The genome of the anaerobic fungus *Orpinomyces* sp. strain C1A reveals the unique evolutionary history of a remarkable plant biomass degrader. Appl Environ Microbiol. 2013;79:4620–34. 10.1128/AEM.00821-13. 23709508 PMC3719515

[bib178] Yu W, Wu Y, Li D. Oxidative cleavage of cellulose by fungi in the termite gut. Int J Biol Macromol. 2025;284:138222. 10.1016/j.ijbiomac.2024.138222. 39622373

